# How volant seabirds benefit from sub-surface predators during multi-predator feeding aggregations

**DOI:** 10.1098/rstb.2025.0199

**Published:** 2026-10-15

**Authors:** Max Licht, Edna Correia, Martin Wikelski, Jens Krause

**Affiliations:** ^1^ Faculty of Life Sciences, Albrecht Daniel Thaer-Institute of Agricultural and Horticultural Sciences, Humboldt-Universität zu Berlin, Invalidenstrasse 42, 10115 Berlin, Germany; ^2^ Cluster of Excellence “Science of Intelligence”, Berlin, Germany, Marchstr. 23, 10587 Berlin, Germany; ^3^ Department Fish Biology, Fisheries and Aquaculture, Leibniz Institute of Freshwater Ecology and Inland Fisheries, Müggelseedamm 310, 12587 Berlin, Germany; ^4^ CE3C Centre for Ecology, Evolution and Environmental Changes & CHANGE – Global Change and Sustainability Institute, Faculdade de Ciências da Universidade de Lisboa, 1749-016 Lisbon, Portugal; ^5^ Department of Migration, Max Planck Institute of Animal Behavior, Radolfzell, Germany; ^6^ Department of Biology, University of Konstanz, Konstanz, Germany

**Keywords:** seabirds, predator prey, interspecies interaction, marine ecology, foraging strategies, commensalism, mutualism

## Abstract

Multi-predator feeding aggregations are common in marine ecosystems and frequently involve seabirds. Often labelled opportunistic ‘scroungers’, volant seabirds derive diverse benefits from the association with sub-surface predators (such as cetaceans, pinnipeds, Teleosts) which can significantly enhance their hunting performance. In this opinion piece, we outline the major mechanisms through which sub-surface predators benefit volant seabirds, based on recent quantitative evidence and hypotheses that warrant further research. The mechanisms we identified are: herding prey to the surface, prey detection (local enhancement), reduction of prey cohesion, access to immobilized prey and the attentional overload of prey. Furthermore, we discuss potential interactions between these mechanisms and mechanism-based predictions which can be tested in the field. We propose that the associations with sub-surface predators are ecologically relevant to the extent that they may influence selection breeding sites. We conclude by discussing methodological advancements that have the possibility to further research in this field and go over open questions they might help to answer in the future.

This article is part of the theme issue ‘Multi-predator feeding aggregations in pelagic waters’.

## 1. Introduction

Volant seabirds are commonly featured in studies of multi-predator feeding aggregations (MPFAs), which are marine phenomena where multiple predator species converge on prey such as fish schools or krill swarms [1–12]. Seabirds are continuously faced with the patchy distribution of their prey and, thus, can benefit from either using MPFAs to locate these patches or foraging within MPFAs. Therefore, these foraging strategies have probably evolved owing to the patchy distribution of prey.

A diverse range of sub-surface predators co-occurs with volant seabirds during these aggregations, including cetaceans, pinnipeds, Teleosts and penguins [1–4,7,13–15]. These predators aggregate around the same fish schools, leading to frequent interspecific interactions. The hunting behaviour of some of these predators potentially provides as a by-product, feeding opportunities for volant seabirds [1,16,17].

In this opinion piece, we outline the mechanisms based on which volant seabirds can benefit from the hunting behaviour of sub-surface predators at MPFAs (figure 1). This is timely because the mechanisms underlying these opportunities and the resulting benefits have been investigated only recently in some detail owing to conceptual and methodological advancements. For example, sub-surface predators can make prey more accessible to surface-feeding birds, which was documented with the help of high-resolution sonar [10] and animal-borne cameras [7]. Sub-surface predators have also been documented to greatly reduce the cohesion of prey groups, thus decreasing their collective defences, which was shown with high definition sub-surface cameras [18] and drone footage [6]. Furthermore, researchers have begun to quantify the benefits to seabirds in terms of increased attack and capture frequencies which result from the association with large sub-surface predators [18,19] and compared these to unassociated feeding as a control.

We focus primarily on volant seabirds but many of the mechanisms discussed here could also benefit seabirds that dive for prolonged periods. Penguins, although seabirds, are considered here within the broader category of sub-surface predators rather than as focal beneficiaries of MPFAs. Because our focus is specifically on benefits arising from interactions with sub-surface predators, we do not consider the extensive literature on local enhancement and information transfer among seabirds, which has recently been reviewed by Monier [20].

**Figure 1.**
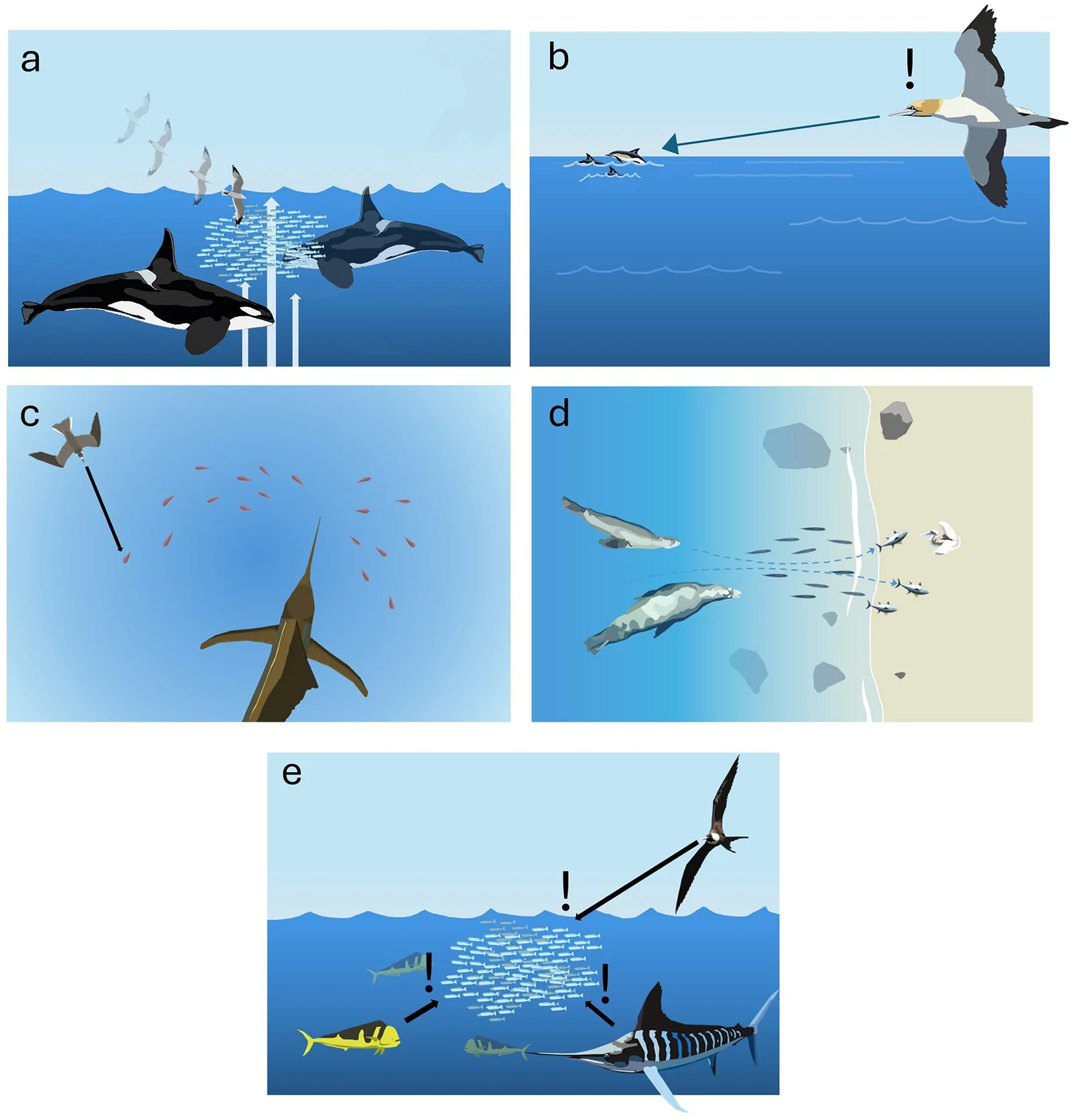
Mechanism through which sub-surface predators benefit seabirds during MPFAs: (a) sub-surface predators herd prey towards the surface and make it accessible; (b) sub-surface predators indicate prey position; (c) predators reduce prey group cohesion, making prey more vulnerable to subsequent attacks; (d) sub-surface predators produce immobilized prey and food scraps; (e) multiple predators split the attention of prey and thereby decrease the efficiency of escape.

While we do not present an exhaustive list, we believe that the mechanisms discussed here are behaviourally important and broadly applicable across many seabird species, although their relative importance is likely to vary among taxa. Moreover, although we have discussed these mechanisms individually, they are likely to act in concert under natural conditions, either sequentially or simultaneously, with consequences that have received little attention to date. Importantly, our understanding of some mechanisms, such as prey cohesion reduction, is now sufficiently advanced to allow predictions about when seabirds are likely to attack and which prey they are likely to target.

The benefits that volant seabirds derive from these interactions may also extend beyond individual foraging events. If interactions with sub-surface predators consistently enhance foraging opportunities, they could potentially influence larger-scale ecological topics, including the selection of migration routes and stopover sites. Further methodological advances that allow these interactions to be observed and quantified at finer temporal and spatial scales will be important for testing these possibilities and addressing the many questions that remain.

## 2. Mechanisms benefiting seabirds when associating with sub-surface predators

### (a) Herding prey towards the surface

Before a prey school can be attacked, it first needs to be accessible. Volant seabirds are limited in their depth reach and some species may not enter the water at all and are reliant on prey that is directly at the surface, such as frigatebirds [1,21,22]. As such, they may be able to herd prey but not from the same depths as sub-surface predators and the latter are one of the primary factors as to why seabirds can prey on pelagic fish schools [1,22,23]. It has been proposed that sub-surface predators perform collective herding of prey towards the surface [1,24], which increases their own hunting performance because it allows them to push the prey against a barrier [25]. For example, sub-surface predators might herd flying fish to the surface where they are caught by red-footed boobies (*Sula sula*) [26,27]. Additionally, some predators might also condense prey schools into tightly packed ‘baitballs’ which might be easier to target [13,28]. Although herding is regularly proposed to be an important mechanism during MPFAs (e.g. [1,16]), most studies on MPFAs only encounter the hunting event after its onset (but see [17,29]). However, there are a few descriptions of predators herding prey to the surface. Nøttestadt *et al.* showed that orcas (*Orcinus orca*) collectively herded schools of herring (*Clupea harengus*) to the surface, seen through hydro-acoustic recordings. Subsequently, the fish were observed to be preyed upon by common gulls (*Larus canus*) and white-tailed eagles (*Haliaeetus albicilla*) [10]. Similarly, African penguins (*Spheniscus demersus*) herd prey fish towards the surface where Cape cormorants (*Phalacrocorax capensis*), sooty shearwaters (*Ardenna grisea*) and great crested terns (*Thalasseus bergii*) and other seabirds joined the hunt, which was observed via animal-borne cameras [7].

In both instances, seabirds joined the hunting event once the prey was near or at the surface, and the authors were thus able to show the emergence of an MPFA. If surface-feeding seabirds are otherwise unable to produce feeding opportunities on their own, or of the same quality, they might depend to a large degree on the presence of sub-surface predators. Once accessible prey is available, seabirds also benefit from associations with sub-surface predators through other mechanisms which are described in the following.

### (b) Detection of prey aggregations

The ability of seabirds to locate prey patches by approaching foraging con- and heterospecifics (local enhancement) is well described and supported by a broad range of literature on inter-bird interactions [20,30]. It is likely that seabirds respond to sub-surface predators along similar mechanisms. Marine predators (e.g. dolphins, tuna, etc.) that target prey schools are usually larger bodied than their prey [31], meaning that, for a given visual acuity, they can be detected by seabirds from greater distances. They also often hunt in groups, which may involve surfacing, breaching and other disturbances of the water surface. The foraging activity of these large sub-surface predators is thus likely to provide a more conspicuous visual cue to seabirds than a prey group that is not being predated on.

It has often been proposed that seabirds show attraction towards feeding sub-surface predators [2,32–35] but this remains to be conclusively demonstrated. In observations of feeding dusky dolphins (*Lagenorhynchus obscurus*), Vaughn *et al.* [24] reported that the number of seabirds at MPFAs increased from the onset of dolphin feeding to 2 minutes into feeding, even when no seabirds were present before the dolphins began feeding.

Seabirds have also been shown to associate with non-feeding sub-surface predators (e.g. [24,34,36]), suggesting that they might follow in anticipation of feeding opportunities that may arise. This would create a different scenario for the onset of MPFAs, in which a pre-existing predator association encounters a prey group collectively rather than different predator species joining an ongoing foraging event consecutively.

### (c) The reduction of prey cohesion

The collective defences of group-living prey are largely dependent on strong cohesion and high density [37,38]. Therefore, many predators attempt to disperse and fragment the school to increase capture success [37,39–41]. Predators often prefer to attack individual prey that are temporarily isolated from the group (e.g. [42–44]) or those with larger distances between them and their neighbours (‘domains of danger’) [45–47]. This means that a predator, which disrupts the prey cohesion through an initial attack, can temporarily create favourable foraging conditions which make subsequent attacks by itself or other predators more successful until the prey group has reformed [6,18,48]. Predators capable of substantial cohesion reduction are usually large bodied (i.e. large enough to disperse enough fish to cause a disruption of the schooling behaviour) and relatively fast moving such as common dolphins (*Delphinus delphis,* [18]) and sailfish (*Istiophorus platypterus* [6]). Thiebault *et al.* [18] showed that the capture likelihood of Cape gannets (*Morus capensis*) increased, if another predator (gannet or dolphin) had attacked the prey school shortly before their own attack. And Licht *et al.* [6] demonstrated that the timing of tern attacks coincided with reductions of prey cohesion shortly after sailfish attacks, which had been predicted in a theoretical study by Lett *et al.* [48].

### (d) Immobilized prey, food scraps and kleptoparasitism

Hunting efficiency of seabirds can be boosted by sub-surface predators limiting the mobility of prey. Galapagos sea lions (*Zalophus wollebaeki*) were observed to herd schools of scad (*Decapterus muroadsi*) towards the shore from the open water. Once they reached the shore, the fish beached on the sand and were easily picked off not only by sea lions that conducted the hunt but also scrounging pelicans (*Pelecanus occidentalis*) [49]. Similar behaviour is also known for some populations of bottlenose dolphins (*Tursiops truncatus*), which, by beaching prey fish, produced foraging opportunities for an avian fish-eating predator (albeit non-seabird), the great egret (*Ardea alba*), whose energy intake per hour increased significantly when foraging with dolphins [50].

Furthermore, some seabirds might not be targeting live prey fish at all during these associations. Instead, they might be targeting the floating food scraps, which are produced during the foraging of the sub-surface predators [34,51]. This type of benefit can lead to the emergence of MPFAs, even in the absence of schooling prey. For example, seabirds associate with grey whales (*Eschrichtius robustus*) when they feed on benthic organisms. The mud they bring up to the surface provides feeding opportunities for the seabirds [52,53].

Another aspect that is related to immobilized prey is kleptoparasitism. Some seabird species might be attracted to MPFAs for the sole purpose of stealing prey from other predators (see [17]). Although this is described primarily between seabird species, some seabirds have been observed to scrounge stunned prey from sub-surface predators. O'Corry-Crowe *et al.* [54] described how glaucous gulls (*Larus hyperboreus*) try to scrounge prey fish from narwhals (*Monodon monoceros*) in the Canadian High Arctic.

### (e) Attentional overload of the prey

The confusion effect is a well-known mechanism which produces anti-predatory benefits for grouping prey [37,55,56]. A predator which is faced with multiple potential targets, has to account for all of their positions and movement trajectories, thereby splitting its attention, leading to reduced attack frequencies and capture probabilities [56–58]. An animal under attack by more than a single predator might similarly be expected to split its attention to account for the position of different predators and their movement trajectories. To our knowledge, it has not been tested whether the confusion effect could potentially work to the detriment of the prey targeted by group-hunting predators. This potential effect may be exacerbated by simultaneous attacks from multiple directions (i.e. horizontally by sub-surface predators and vertically by seabirds). By splitting their attention, the prey animals are expected to react slower to approaching predators and choose suboptimal flight paths, which should increase the capture probability for predators. In line with this idea, Lett *et al.* [48] created a model showing that successive attacks by multiple predators, especially predators attacking from different directions such as gannets and dolphins, create higher disturbance and lower cohesion within prey groups.

## 3. Interactions between mechanisms and mechanism-based predictions

The mechanisms described above are unlikely to operate independently. Some may be prerequisites for others and some may occur simultaneously or reinforce one another, making it difficult to attribute an observed foraging benefit to a single mechanism. For example, herding by large sub-surface predators can bring prey to the surface, making them accessible to volant seabirds and enabling subsequent mechanisms such as prey cohesion reduction and increased prey vulnerability to take effect. Similarly, multiple simultaneous attacks may generate attentional overload in prey, potentially reducing cohesion or further increasing the vulnerability of already isolated individuals. In this context, modelling by Rahmani *et al.* [59] suggests a trade-off between the attention individuals allocate to monitoring nearby group members, which is necessary to maintain cohesion, and attention allocated to factors outside the group, such as monitoring predators. Such interactions could therefore produce additive or synergistic effects among mechanisms, but their relative contributions are likely to be difficult to disentangle. Currently, very little is known about the interaction between mechanisms, which calls for both more modelling and empirical studies on this topic.

Nevertheless, the temporal and spatial dynamics of prey responses can sometimes allow mechanism-specific predictions to be made. For example, the extent and duration of prey cohesion reduction following an attack by a large-bodied predator can be quantified and related to the timing of subsequent seabird attacks [6]. If seabirds preferentially target prey that have become isolated following such attacks, detailed observations could therefore be used to predict both when an attack is likely to occur and which individuals are most likely to be targeted. Similarly, after orca slapped their tail into herring schools to stun individuals [60], the prediction would be that gulls focus not on those herring closest to them but on those that were immobilized even if they are further away than active fish. This means that detailed knowledge of the above mechanisms can result in mechanism-specific predictions which can be tested in field and laboratory experiments and deepen our understanding of which mechanisms are at play and which specific benefits they produce.

Identifying what volant seabirds might do in the context of a particular benefit is one level of prediction. Another is to investigate whether volant seabirds are capable of anticipating the hunting strategies of sub-surface predators. For example, brown pelicans associated with sea lions that repeatedly drive fish onto beaches in the Galápagos may potentially anticipate where prey will be stranded and adjust their movements accordingly [49]. Distinguishing such anticipation from simple following behaviour would require observations of seabird movements before the outcome of the predator's hunt. More generally, this raises the possibility that seabirds could use information about predator behaviour to predict where and when foraging opportunities will arise.

## 4. How volant seabirds might benefit sub-surface predators

The mechanisms outlined above demonstrate how seabirds benefit from associating with sub-surface predators, and why their hunting performance can improve in MPFAs (figure 1). Importantly however, seabirds are not always merely scroungers in these interactions but they can also generate benefits for other predators. This is most clearly documented in the context of local enhancement, which has been studied in both intra- and interspecific seabird flocks (e.g. [16,30,61]). However, the ability of seabirds to locate prey and indicate its presence might not be exclusively used by other volant seabirds. For instance, Sutton *et al.* [62] suggest that little penguins (*Eudyptula minor*) might use volant seabirds as indicators of prey location. It seems likely that the diving behaviour of large-bodied seabirds such as gannets or pelicans should provide acoustic cues which can be heard over long distances and which might attract sub-surface predators such as dolphins or sharks. Nevertheless, this has yet to be tested.

Some seabirds can also herd prey schools. This behaviour is most pronounced in species capable of extended dives, such as alcids, and can create feeding opportunities for other predators [16,63,64]. Anderwald *et al.* [63] reported that minke whales (*Balaenoptera acutorostrata*) exploit such situations, benefitting from the foraging efforts of common murres (*Uria aalge*) and other diving seabirds.

Additionally, any predator feeding on fishes is likely to generate food scraps [16,51], which could potentially benefit small bird species. Large seabirds, such as brown pelicans, may also disrupt prey school cohesion during their attacks and benefit other predators [39]. While this potential effect has not yet been empirically tested, it remains uncertain whether sub-surface predators are mobile enough to capitalize on the brief windows of reduced cohesion caused by seabirds. Furthermore, if the attentional overload discussed in §2e is biologically relevant, it would probably benefit all predators, regardless of whether they are seabirds or sub-surface predators.

## 5. Ecological relevance of multi-predator feeding aggregations for seabirds

Volant seabirds can benefit during MPFAs through various mechanisms as shown above. Their hunting efficiency can improve both through enhanced benefits, such as higher prey capture probability [18], and reduced costs, such as lower movement expenditure [65]. Participating in MPFAs can therefore represent a beneficial foraging strategy. While it is unlikely that any seabird species feed exclusively in association with other sub-surface predators, some species may rely heavily on these associations or show a strong preference for them [19,22].

Feeding at MPFAs may be especially important during energetic bottlenecks. One such bottleneck may occur during breeding, when energy demands increase sharply because offspring needs to be fed. Associating with sub-surface predators might become more important during these periods. Reynolds *et al.* described long-term shifts in the diet of a breeding colony of sooty terns (*Onychoprion fuscatus*) over multiple decades. They observed a steep decline in the number of breeding pairs by 84% [66], which coincided with the onset of tuna fisheries around the colony [21]. They suggest that the presence of tuna and other large predators is crucial for some seabird species during breeding as they produce beneficial foraging conditions for seabirds [21,22,67].

A second major bottleneck might be migration. To sustain long migratory journeys, individuals must forage efficiently. Marine areas with big populations of large sub-surface predators may be particularly attractive to migrating and wintering birds, as they might provide foraging benefits of MPFAs as outlined above [19,68]. For example, Correia *et al.* [19] showed that several tern species regularly exploited MPFAs at an important non-breeding and stopover site in the Bijagós Islands, substantially boosting their foraging performance. This suggests that the presence of large sub-surface predators may be an overlooked factor influencing seabirds’ migration route and stopover site selection, as these predators can create favourable foraging opportunities for many seabird species.

## 6. Methodological advancements

### (a) Tags and biologgers

Biologgers on animals initially only recorded simple parameters of animal movement (but see [69,70]). Since about a decade ago, however, highly advanced devices and novel on-board analytical methods have been employed to provide sophisticated information on individual and group behaviour as well as the environmental conditions of free-ranging animals in marine systems [71]. To quantify whether seabirds may benefit from MPFAs, their individual foraging effort and foraging success first needs to be assessed remotely. A pioneering study showed that changes in stomach temperature can detect individual feeding events [72]. Furthermore, animal-borne data loggers and video cameras can verify predator–prey interactions, as well as social interactions [73].

Going a step further in quantifying interactions directly, it is also possible to use acoustic telemetry tags, for example, on fishes, whose telemetry signals are received by GPS data loggers on their predators [74]. A similar approach can be used when including shipping radar detectors in seabird data loggers [75] or Bluetooth proximity detectors on several birds outfitted with biologgers [76]. Such approaches have now become possible because of energy savings by tight time synchronization [77]. If several birds are reporting back from the same detectors at the same time, a feeding aggregation can be inferred. In the near future, intelligent onboard algorithms will be able to detect when an individual starts to forage and then start, or increase the sampling frequency, of other sensors such as GPS, three-dimensional acceleration (detecting dives, or micro-turbulence for other seabirds), magnetometer, pressure sensors or humidity sensors as well as acoustic monitors [78]. The data fusion—that is, combining data from multiple sensors and individuals—possible now also onboard [79], as well as pre-trained artificial intelligence algorithms can then identify whether an individual is part of an intra- or interspecific feeding aggregation, how long it spends in such an aggregation and whether its success is higher compared to solitary foraging events. Ideally, the sensor settings as well as the input into the detection algorithms should be calibrated and verified by direct behavioural observations, ideally involving drones.

The use of tags and biologgers to study MPFAs could be further developed by combining them with visual monitoring using animal-borne cameras or drones, allowing MPFA-specific patterns in biologging data to be identified.

### (b) Drones

Unmanned aerial vehicles (UAVs) offer the possibility to monitor and observe seabirds from above while being close enough to distinguish individuals and observe their behaviour [80,81]. While drones have been widely applied in monitoring populations and breeding success [80], drones can also be used to study the foraging behaviour of seabirds [6,82–84]. Compared to traditional boat-based surveys [85], drone-based filming offers several advantages. The top-down perspective can provide insights into the visual cues which seabirds use when making foraging decisions, such as prey distribution and behaviour [6], or the features of preferred feeding sites [83,84]. UAV footage also enables fine-scale spatial tracking, including the reconstruction of flight paths which can reveal, for example, search behaviour. In addition, drones make it possible to monitor multiple individuals simultaneously when they forage in proximity, and to quantify behaviours that are otherwise difficult to measure in the field, such as wing-beat frequency. It also allows the monitoring of multiple metrics at the same time, which might be otherwise impossible to assess.

### (c) Animal-borne cameras

Animal-borne cameras offer the opportunity to obtain visual information on the environment of an animal over long periods of time, which otherwise might be impossible to access, especially for animals of the open ocean (e.g. [86,87]). In the context of MPFAs, they have the potential to reveal previously unknown interactions with conspecifics or other species [88–90], and can help explore the frequency of co-occurrences of different predator species during foraging [91,92]. For example, Sutton *et al.* [62] observed short-tailed shearwaters (*Puffinus tenuirostris*) flying towards prey aggregations in the footage of a camera attached to a little penguin (*E. minor*), shortly before it swam in the same direction and encountered that prey group, suggesting that it might have followed the shearwaters. Animal-borne cameras can also serve to ground-truth behavioural coding of GPS flight tracks [93]. Revealing the animal's point of view over entire foraging trips, they can also show how MPFAs originate [7] and how many foraging events might be encountered in a single trip. In the future, animal-borne cameras could produce data on the frequency and reliance of foraging in association with other predators through videographic evidence.

### (d) Marine radar

MPFAs can be very hard to find given the vast areas over which seabirds and sub-surface predators are active. In recent years, the use of fishing boat radar has been used to assess the spatial distribution of seabirds and their trajectories [94,95]. Originally used to study how seabirds use each other to increase search efficiency, this use of radar might also help with the localization of MPFAs. This could considerably facilitate the study of MPFAs because less time is spent on ‘blind’ searching, driving transects and visual searches for bird flocks (which only work over relatively limited spatial distances).

## 7. Open topics and questions

We believe that research on MPFAs is approaching a new phase given the recent conceptual and methodological developments. Foraging benefits for volant seabirds gained through the association with sub-surface predators are receiving increasing quantitative evidence in form of attack and capture rates which are also measured when these bird species engage in unassociated foraging for comparison. Furthermore, the identification of mechanisms by which seabirds can benefit from sub-surface predators is beginning to be detailed enough for predictions as to when birds can benefit and which prey they should target. Our understanding of how seabirds benefit from sub-surface predators is thus moving from the descriptive to the predictive. This shift will also allow us to attribute benefits (at least in some cases) to specific mechanisms and to quantify their importance. At the same time, an understanding of the mechanisms opens the door to a broader ecological perspective, allowing a link to ecosystem-level consequences. With this expanding set of methodological possibilities and conceptual developments, it is timely to consider the next steps for the field, outlining pressing open questions, and exploring new approaches for investigating multi-species feeding aggregations:

— relatively little is known about how different mechanisms (which occur as a by-product of sub-surface predators hunting on fish schools) interact with one another. This is a problem which can be addressed through modelling in combination with fieldwork testing modelling predictions;
— seabirds in general seem to almost universally profit from associations with other predators, but do they in turn produce benefits for sub-surface predators as well?
— how do seabirds and other predators locate MPFAs? Which cues do they use and how does this differ between different predator species? For example, could the diving behaviour of large-bodied seabirds such as gannets provide acoustic cues which may be used by dolphins and sharks to locate fish schools?
— are there seabirds or other predators that are attracted to MPFAs solely to prey on other predator species?
— given that tags on birds will increasingly allow us to obtain time budgets for associated and unassociated feeding, we should be able to place seabird species on a continuum of how reliant they are on sub-surface predators for their foraging behaviour;
— during which time period (e.g. incubating, chick rearing, migrating, overwintering) is the dependence on the associations with sub-surface predators the greatest?
— can the absence of large sub-surface predators, for example, through the onset of targeted fishing, cause the reduction or even extinction of seabird colonies?
— can the return or recovery of populations of major sub-surface predators, such as large Teleosts or cetaceans, revive seabird colonies because tuna will provide substantially improved foraging opportunities for some seabird species?
— what are the costs of associated feeding? Hunting alongside other predators may also come with costs which need to be quantified in relation to the benefits. Furthermore, the mechanisms we described may only provide benefits to volant seabirds up to a certain group size, beyond which costs start to outweigh the benefits. This topic needs further research in the context of species-specific costs and benefits of foraging association with sub-surface predators;
— to what extent do tropical, temperate and polar seabirds differ in their reliance on MPFAs, and how does sub-surface predator composition vary across latitudinal and productivity gradients, given that large Teleosts appear to be the primary instigators in tropical systems while marine mammals may assume this role at higher latitudes? and
— to which degree have some seabird species evolved specific adaptations to feed during MPFAs? Are there discernible differences (behavioural, morphological) between species that rely on MPFAs and species that do not (or much less so)?

## Data Availability

This article has no additional data.

## Theme

One contribution of 14 to a theme issue ‘Multi-predator feeding aggregations in pelagic waters’.
